# Effects of compost amendments and experimental drought on grassland soil microbial communities

**DOI:** 10.1093/femsle/fnaf108

**Published:** 2025-10-06

**Authors:** Daniela Guasconi, Gustaf Hugelius, Karina E Clemmensen, Sara A O Cousins, Jaanis Juhanson, Stefano Manzoni, Nina Roth, Petra Fransson

**Affiliations:** Department of Physical Geography and Bolin Centre for Climate Research, Stockholm University, 106 91 Stockholm, Sweden; Department of Physical Geography and Bolin Centre for Climate Research, Stockholm University, 106 91 Stockholm, Sweden; Department of Forest Mycology and Plant Pathology, Uppsala BioCenter, Swedish University of Agricultural Sciences, 750 07 Uppsala, Sweden; Department of Physical Geography and Bolin Centre for Climate Research, Stockholm University, 106 91 Stockholm, Sweden; Department of Forest Mycology and Plant Pathology, Uppsala BioCenter, Swedish University of Agricultural Sciences, 750 07 Uppsala, Sweden; Department of Physical Geography and Bolin Centre for Climate Research, Stockholm University, 106 91 Stockholm, Sweden; Department of Physical Geography and Bolin Centre for Climate Research, Stockholm University, 106 91 Stockholm, Sweden; Department of Forest Mycology and Plant Pathology, Uppsala BioCenter, Swedish University of Agricultural Sciences, 750 07 Uppsala, Sweden

**Keywords:** drought, compost, microbial communities, grassland

## Abstract

Prolonged drought is a major stressor for grassland ecosystems. In addition to decreasing plant productivity, it can affect soil microbial activities and thus destabilize nutrient cycling and carbon (C) sequestration. Soil organic amendments (OAs), such as compost, can be used to enhance soil fertility and mitigate drought effects. In this study, we evaluated the responses of fungal and bacterial communities to a 3-year-long experimental drought and compost treatment across four soil depths in two Swedish grasslands and at an upper and a lower topographic position. Results showed that while drought reduced soil moisture and compost amendment increased C content in the topsoil, the effects on microbial abundance and community composition within this time frame were weak, and detectable only in the topsoil. Fungal abundance increased with compost addition, which also affected community composition, while fungal communities were resistant to drought. Bacterial communities were not significantly affected by any of the treatments. This suggests that microbial ecosystem functions were resistant to the experimentally reduced precipitation. Overall, variation between sampling sites was more important for microbial community composition than treatments, highlighting the need for a better understanding of small-spatial-scale environmental controls on soil microbial and plant communities and their ecosystem functions.

## Teaser

A 3-year study on Swedish grasslands found that compost amendments increased topsoil fungal abundance in the soil, but fungal and bacterial communities were resistant to drought.

## Introduction

Drought, defined as a prolonged period with precipitation below average (Dai 2011), can impose a significant stress on grassland ecosystems, including reduced productivity and altered plant species composition, with potential effects on biogeochemical cycling (Wu et al. 2011, Chavez Rodriguez et al. 2023). As dry periods lengthen and extreme weather events become more frequent (Knapp et al. 2008, IPCC 2021), research has focused on understanding the impact of reduced precipitation and temporal fluctuations in rainfall on plant productivity. Soil microbial communities are important drivers of ecosystem processes such as nutrient cycling and organic matter decomposition (Schimel 1995, Sowerbya et al. 2005), and therefore play a key role in maintaining ecosystem functionality in grasslands. Given their sensitivity to environmental change, alpha and beta diversity of fungi and bacteria can also be important indicators of ecosystem functioning (Yang et al. 2021). Changes in environmental conditions can alter community composition by selecting for organisms whose ecological niches and physiological tolerances match the prevailing conditions. Taxa whose niches are poorly aligned with the new environment may decrease in abundance or go dormant (Vos et al. 2013), whereas generalist or more resilient taxa with broader ecological niches may thrive (Evans and Wallenstein 2013), thereby favoring the growth of particular microbial functional groups. Microbial communities experience continuous disturbance on short temporal scales, including cycles of drying and rewetting related to precipitation events (Schimel et al. 2007). Even if microbial communities are adapted to these cycles, prolonged drought stress can lead to shifts in microbial abundance (Sun et al. 2020) and community composition (Evans and Wallenstein 2013), reduced metabolic activity (Franco-Andreu et al. 2017, Bai and Cotrufo 2022), and decreased bacterial diversity (Preece et al. 2019), but also improved capacity to recover after drying-rewetting events (Leizeaga et al. 2022). The existence of complex above-belowground feedbacks between plant communities and their associated microbial communities (van der Heijden et al. 2008, Bever et al. 2013), such as the positive association between the species richness of arbuscular mycorrhizal fungi and grassland plant species (Wardle et al. 2004, Van Der Heijden et al. 2006, Hiiesalu et al. 2014), may in turn have implications for grassland properties and functionality. Grassland functions such as primary productivity and carbon storage further support ecosystem services ranging from forage production to climate mitigation, which are key for reaching Sustainable Development Goals (SDG) 13 Climate Action and 15 Life on Land.

In recent decades, increasing soil organic matter content has been identified as a key objective for sustainable agricultural production and to address soil degradation issues, such as erosion and nutrient depletion (Doran and Zeiss 2000, Stott and Moebius-Clune 2017), which are exacerbated by water scarcity (Cárceles Rodríguez et al. 2022). This includes managing negative effects of drought on grasslands and croplands, as reduced plant productivity poses a direct threat to food security worldwide (Cárceles Rodríguez et al. 2022) (SDG 2 Zero Hunger). Soil organic amendments (OA) such as compost, biochar, or manure have proven useful to address some of these challenges and to mitigate negative effects of drought. These amendments increase nutrient and organic matter input to the soil, which stimulate plant growth (Ryals and Silver 2013) and microbial activity (Franco-Andreu et al. 2017, Luo et al. 2018, Hammerschmiedt et al. 2021), and increase microbial biomass (Bastida et al. 2017, Luo et al. 2018, Hammerschmiedt et al. 2021, Sarker et al. 2022). This, in turn, enhances soil aggregation by stimulating the growth of fungal hyphae and plant roots that can physically retain soil particles (Sarker et al. 2022), and through the mycorrhizal production of extracellular compounds that bind soil particles (Wu et al. 2024), reinforcing overall soil structure and stability and counteracting soil erosion. OA may also increase microbial carbon use efficiency (CUE, Gravuer et al. 2019, Rocci et al. 2021), which, in turn, improves C stabilization via the so-called “*in vivo*” stabilization pathway by promoting C retention in microbial biomass (i.e. stabilization of microbial necromass; Liang et al. 2017). Overall, the resulting increase in soil organic matter (SOM) also leads to improved soil water-holding capacity, thus buffering soil moisture loss during dry periods (Hueso et al. 2011).

The organic amendments may also affect microbial community composition, e.g. by favoring the growth of specific microbial groups such as saprotrophic decomposers, which thrive in environments rich in easily accessible C (Dai et al. 2018). This addition of labile C compounds potentially increases the decomposition rate of native organic matter via the so-called priming effect. Some microbial groups such as plant pathogens may be suppressed by OA (Jaiswal et al. 2017, Dundore-Arias et al. 2020), whereas arbuscular mycorrhizal growth may be stimulated (Raghuwanshi and Upadhyay 2004, Jiang et al. 2021). These differential responses suggest that a focus on microbial functional groups might provide better insights into the effects of drought or OA on ecosystem functions in grasslands such as plant growth and soil nutrient cycling, compared to studies that solely focus on total microbial abundance. Given the importance of microbial metabolism in C cycling, changes in community composition may also have positive or negative feedbacks on soil organic C accumulation, which should be accounted for in soil organic C management designs. For instance, a higher abundance of arbuscular mycorrhizal fungi is associated with higher proportion of organic matter stored as mineral-associated organic matter, which turns over slowly and thus allows longer-term C sequestration compared to soils with low mycorrhizal abundance (Bai and Cotrufo 2022).

The effects of changing climatic conditions and land management on microbial communities and soil functions are not simply additive. For example, soil properties and climate interactions modulate bacterial and fungal responses to rewetting (Li et al. 2023). These interacting effects require multifactorial field experiments to be understood, as laboratory observations are generally too short to mimic natural ecosystem change and can lead to biased results by artificially selecting for specific microbial functional groups (Canarini et al. 2017) and by disrupting soil structure when preparing the samples for incubation. Therefore, laboratory studies may not detect spatial variability within the landscape, if the effects of such variability on the measured properties are masked by the effect of soil preparation. Soil communities also vary with soil depth, with microbial communities residing in the topsoil experiencing highest soil moisture fluctuations and receiving most organic matter inputs. By contrast, subsoils are less affected by climate and more dependent on edaphic properties (Mathieu et al. 2015), and SOM formation in subsoils is less dependent on fresh plant litter input, and more linked to root inputs and microbial processing (Cotrufo et al. 2021). These differences may also depend on physical properties such as soil texture and type. Because of these multiple and interacting environmental and soil drivers, it is difficult to predict at what depth climate or OA may exert an effect on soil microbial communities.

Changes in climate and land management may cause both transient or long-lasting shifts in plant and microbial community composition, potentially impacting ecosystem processes and functions. While OA can increase SOM content and nutrient availability and mitigate drought effects in grasslands, optimizing sustainable agricultural practices requires a better understanding of their effects on soil microbial communities. In this study, we evaluate the response of fungal and bacterial communities in two Swedish grasslands (each with plots at different locations along a hillslope) after four seasons of compost amendment and simulated drought, either as single or combined treatments. The microbial communities are studied at four different depths (0–10 cm, 10–20 cm, 20–30 cm, and 40–50 cm), to assess the impact of the treatments in different soil layers.

We hypothesize that compost addition and drought will shift fungal and bacterial community composition and the relative abundance of fungal functional groups and have contrasting effects on microbial abundance and diversity. Specifically, we expect that (I) drought will decrease overall fungal and bacterial abundance and diversity, but (II) compost amendments will increase microbial abundances with a relatively larger increase in saprotrophic and mycorrhizal fungi, and compost will further mitigate the loss of soil moisture and microbial alpha diversity. Finally, (III) the effects of drought and compost will be strongest in the topsoil, where soil moisture is expected to decrease the most under drought and where organic matter inputs are concentrated.

## Materials and methods

### Site description and experimental setup

The study was carried out close to Tovetorp Research Station in Sörmland, south-east Sweden, on two clay-rich former arable fields converted into grasslands, Tovetorp and Ämtvik (described in Roth et al. 2023, Guasconi et al. 2023). In each grassland, one site was set up at a higher slope position and one at a lower position, about 50 m from each other, with an elevation difference of not more than 6 m. The lower sites have higher soil moisture at deeper depths (+10% volumetric soil moisture below 60 cm depth). Both grasslands are characterized by perennials, but a forest border with a mixed deciduous and conifer forest is closer in Ämtvik (6–20 m) compared to Tovetorp (50–90 m). In each of the four sites, three replicate plots (2×2 m) were set up for each of the four treatments: compost addition, experimental drought, drought and compost combined, and untreated controls, for a total of 48 plots. The drought treatment started in July 2019 with the construction of rain-out shelters (3 per site, 12 in total) designed according to the recommendations of the Drought-Network (Knapp et al. 2017, Yahdjian and Sala 2002). The rain-out shelters are made of evenly placed, v-shaped polycarbonate strips, which exclude 60% of the precipitation from April to November. Each rain-out shelter covered one compost-free plot (drought treatment) and one compost plot (drought-compost treatment). The compost used for the soil amendment was prepared from green parts of *Zea mays* harvested in 2019. The leaves and stems were cut into small pieces, piled, and regularly mixed for ca. four months. The compost obtained from the *Z. mays* residues had a C:N ratio of 9.8 and was applied at a rate of 11 kg m^−2^ in mid-February 2020, as described in Guasconi et al. (2025), following Ryals and Silver (2013).

### Soil sampling and analyses

Soil samples were collected in August 2022 at four depths (0–10 cm, 10–20 cm, 20–30 cm, and 40–50 cm), from one core per plot. Precipitation during August 2022 was close to the average of the previous 10 years (39.2 mm), with one moderate precipitation event one week before the sampling started, whereas air temperature was on average 16.7°C (1.9°C above the average for the month of August; http://weather.zoologi.su.se). Samples for total soil C and N measurements and DNA analyses were collected using a Pürckhauer soil corer (2.5 cm diameter; Eijkelkamp, The Netherlands). The 1 m long soil core was split in sections in the field, frozen immediately after collection, and later freeze-dried and ground with a mortar before analyses. Samples for bulk density, soil organic matter (SOM, calculated through loss on ignition at 550°C), soil pH, and soil nutrients (P, Ca, Mg, K) measurements were collected with a root auger (8 cm diameter; Eijkelkamp, The Netherlands) within 50 cm from the first core. Total soil C and N and soil C stocks (kg C/m^2^) were calculated from elemental analyses carried out by the Stable Isotope Facility at UC Davis (California), soil bulk density, and SOM measures. As soil pH was around 6, total C is interpreted here as organic C. Soil moisture measurements were conducted every 3 weeks during the entire growth period (2019–2022) in each plot, using a PR2 profile probe (Delta-T Devices Ltd, Cambridge, UK). The analyzed data consists of growing season averages of volumetric soil water content (%) at the four sampling depths. Root biomass was collected in August 2022 with a root auger (8 cm diameter; Eijkelkamp, The Netherlands) as described in Guasconi et al. (2025), through 5 cm thick soil samples taken to a depth of 30 cm in all plots. Fresh roots were first cleaned from soil on a 0.5 mm mesh and then scanned to estimate root length and diameter using WinRhizo (Regent Instruments, Québec, CA). These measures were used to calculate root traits such as mass density (RMD, g_roots_ cm^−3^_soil_), specific root length (SRL, cm g^−1^_roots_), and root tissue density (RTD, g_roots_ cm^−3^_roots_). After that, the roots were dried at 60°C for 48 h to obtain dry weight.

### DNA extraction, quantification, and sequencing of fungi and bacteria

DNA extraction, quantification, and sequence processing followed the protocol in Hoeber et al. (2021), with modifications described in Guasconi et al. (2023) for the deep soil samples (40–50 cm depth). Estimation of bacterial and fungal community abundances was done by quantifying the 16S rRNA gene and the ITS2 region, respectively, with duplicate runs of quantitative PCR (qPCR), as in Guasconi et al. (2023). The resulting gene copy numbers were adjusted per gram of dry soil in the original sample the DNA was extracted from, and used to calculate the fungal-to-bacterial ratio. The 16S rRNA gene and ITS region were amplified through PCR to obtain bacterial and fungal libraries. The primers used for fungi were gITS7 (forward; GTGARTCATCGARTCTTTG; Ihrmark et al. 2012), ITS4 (reverse; 75%; 5′-TCCTCCGCTTATTGATATGC-3′, White et al. 1990), and ITS4a (reverse; 25%; 5′-TCCTCGCCTTATTGATATGC-3′, Sterkenburg et al. 2015). The primers used for the bacteria were Pro341F (forward; CCTACGGGNBGCASCAG) and Pro805R (reverse; GACTACNVGGGTATCTAATCC; Takahashi et al. 2014). The bacterial PCR followed the same procedure as in Guasconi et al. (2023), and the fungal PCR as in Hoeber et al. (2021). Cycle numbers were kept as low as possible to minimize length biases among fungal species (Castaño et al. 2020). The final PCR products were pooled in equal DNA quantities for sequencing. Adaptor ligation and sequencing were performed by NGI-Uppsala/SciLifeLab (National Genomics Infrastructure, Uppsala, Sweden) using one PacBio Sequel SMRT cell (v3) (Pacific Biosciences, Menlo Park, California, USA) for the fungal pool, and using the MiSeq platform with the 2 × 250 bp paired-end chemistry (Illumina, San Diego, CA, USA) for the bacterial pool.

### Sequence processing and taxonomic classification

ITS2 reads obtained from the sequencing were processed and clustered following Kyaschenko et al. (2017) through the SCATA bioinformatics pipeline (https://scata.mykopat.slu.se) and classified into operational taxonomic units (OTUs) based on single-linkage clustering with a 1.5% dissimilarity threshold. Identification was done with BLASTn on the PlutoF platform (Abarenkov et al. 2010) in the UNITE database (fungal database, version 8.4). Representative sequences from each OTU were matched to global species hypotheses (SH) in UNITE (Kõljalg et al. 2013). Fungal OTUs were assigned to taxonomic groups according to representative sequence similarity (Guasconi et al. 2023). After quality control and removal of non-fungal OTUs and of OTUs with 3 counts or fewer, the fungal dataset consisted of 1138 OTUs (127 955 sequence counts). Identified species were then assigned to fungal functional groups using the FungalTraits database (Põlme et al. 2020), through which 718 OTUs (approximately 80% of the total fungal sequences) were assigned to saprotrophic, pathogenic, or mycorrhizal functional groups (Guasconi et al. 2023), leaving the remaining taxa as “unknown.” For calculating diversity indexes, the fungal dataset was rarefied by scaling with ranked subsampling (Beule and Karlovsky 2020) to 188 reads per sample.

16S reads were processed using FASTX-toolkit (http://hannonlab.cshl.edu/fastx_toolkit) and merged using PEAR (Zhang and Kobert 2014). Sequences were clustered into OTUs with VSEARCH (2% dissimilarity threshold; Rognes et al. 2016) and aligned and taxonomically classified with SILVA Incremental Aligner (SINA) referencing the SILVA 138 database (Yilmaz et al. 2014). After removing clusters with fewer than 2 reads across all samples as well as reads from mitochondria, chloroplasts, and eukaryotes, the bacterial dataset consisted 6183 OTUs (4 528 157 sequence counts). The bacterial dataset was rarefied to 11 912 reads per sample. All identified fungal and bacterial phyla and fungal functional groups are presented in Supplementary Table S1, together with their relative abundances. Species accumulation curves were generated for all samples (Supplementary Fig. S1). Fungal and bacterial sequences are available in the European Nucleotide Archive (https://www.ebi.ac.uk/ena/browser/home) under the accession number PRJEB89525.

### Statistical analysis

Effects of the treatments on soil moisture, soil organic C, SOM, roots, and on the ratio of fungi to bacteria were tested with mixed effects models (R package: lme4, Bates et al. 2015), which included compost, drought, and depth as fixed factors and plot (nested within site, defined as one of the four combinations of grassland and slope position) as random factor. Differences in soil moisture between treatments were calculated seasonally (spring: April to June; summer: June to September) and over the entire growing season (April to October). The effect of the treatments on fungal ITS and bacterial 16S copy numbers obtained through qPCR (hereafter called “abundance”) was tested using an aligned rank transformed ANOVA (R package: ART, Wobbrock et al. 2011). The ratio of fungi to bacteria was calculated as the ratio between ITS2 and 16S abundance. Differences in the relative abundance of fungal functional groups and fungal and bacterial phyla between treatments were tested with the mvabund function (R package: mvabund, Wang et al. 2021), which fits generalized linear models to each group. Differences in microbial community composition (data Hellinger-transformed) between treatments were tested using the ANOSIM (Analysis of Similarity) and PERMANOVA (permutational multivariate analysis of variance, which also included depth and site as predictors) functions in R (R package: vegan, Oksanen et al. 2020), and a Mantel test was run to test the relationship between the community composition of major fungal functional groups and soil organic C. These dissimilarities were further visualized using NMDS based on a Bray–Curtis dissimilarity matrix (metaMDS function, R package: vegan, Oksanen et al. 2020). Correlations between fungal or bacterial communities, categorical variables (grassland, slope position, depth, drought, compost), and continuous variables (specific root length, SOM), were assessed using redundancy analysis (RDA) and canonical correlation analysis (CCA) with forward selection and Monte Carlo permutations, with the most appropriate test chosen depending on the data distribution. For these analyses sequencing output per sample was accounted for by including sequence counts per sample as a co-variate. Seven fungal samples were missing out of the total 192. In the RDA analyses, these were replaced with mean values from the other replicates of the same treatment (Guasconi et al. 2023). Indicator Species Analysis (ISA) was conducted to identify species significantly associated with the treatment groups. Indicator values, combining species’ relative abundance and frequency, were tested for significance with 999 permutations using the indicspecies package in R (De Cáceres and Legendre 2009). Differences in fungal and bacterial OTU numbers (hereafter “species richness”) and Shannon–Wiener diversity index (“H”, hereafter “diversity”) between treatments, calculated from rarefied fungal and bacterial datasets, were tested with ANOVAs followed by Tukey’s HSD test, and correlations between species richness and diversity and soil organic C and soil moisture were tested with Pearson correlation tests. The statistical analyses were performed using CANOCO 5.02 (RDA and CCA, Microcomputer Power, Ithaca, New York, USA) and R 4.0.4 (all other tests, R core Team 2021), and residuals were checked graphically.

## Results

### Effects of drought and compost amendment on soil properties and root parameters

The experimentally imposed drought reduced soil moisture over the growing season by an average of 16% in the upper 30 cm (*P* = 0.02), but did not have a significant effect in the deepest soil layer (40–50 cm). The effect of the drought treatment was consistent over sites, years, and seasons (spring, summer, or entire growing season). The compost amendments increased total soil organic C from 2.99% ± 1.03% to 3.53% ± 0.75% (18% increase, *P* = 0.03) and total soil N from 0.24% ± 0.06% to 0.28% ± 0.06% (17% increase, *P* = 0.01) in the topsoil (0–5 cm), with no significant changes to the C:N ratio in any of the soil layers. Even though average soil moisture was slightly lower (by 5.3%) in the plots with combined compost addition and drought compared to the unamended drought plots, the difference was not significant, and overall soil moisture was not affected by the compost treatment. Neither treatment had any significant effects on soil P, Ca, Mg, K, or pH. Drought and compost did not affect root biomass, but drought increased RTD (*P* = 0.048), SRL of fine roots (*P* = 0.049), and average root diameter (*P* = 0.045). In the top 10 cm, we also observed a decrease in SRL of coarse roots under drought (*P* = 0.04) and an increase in RTD (*P* = 0.02) and SRL of all roots (*P* = 0.01) after compost addition. SRL was also correlated strongly with microbial community composition (Supplementary Fig. S2).

### Effect of drought and compost on microbial abundance and community composition

**Figure 1.**
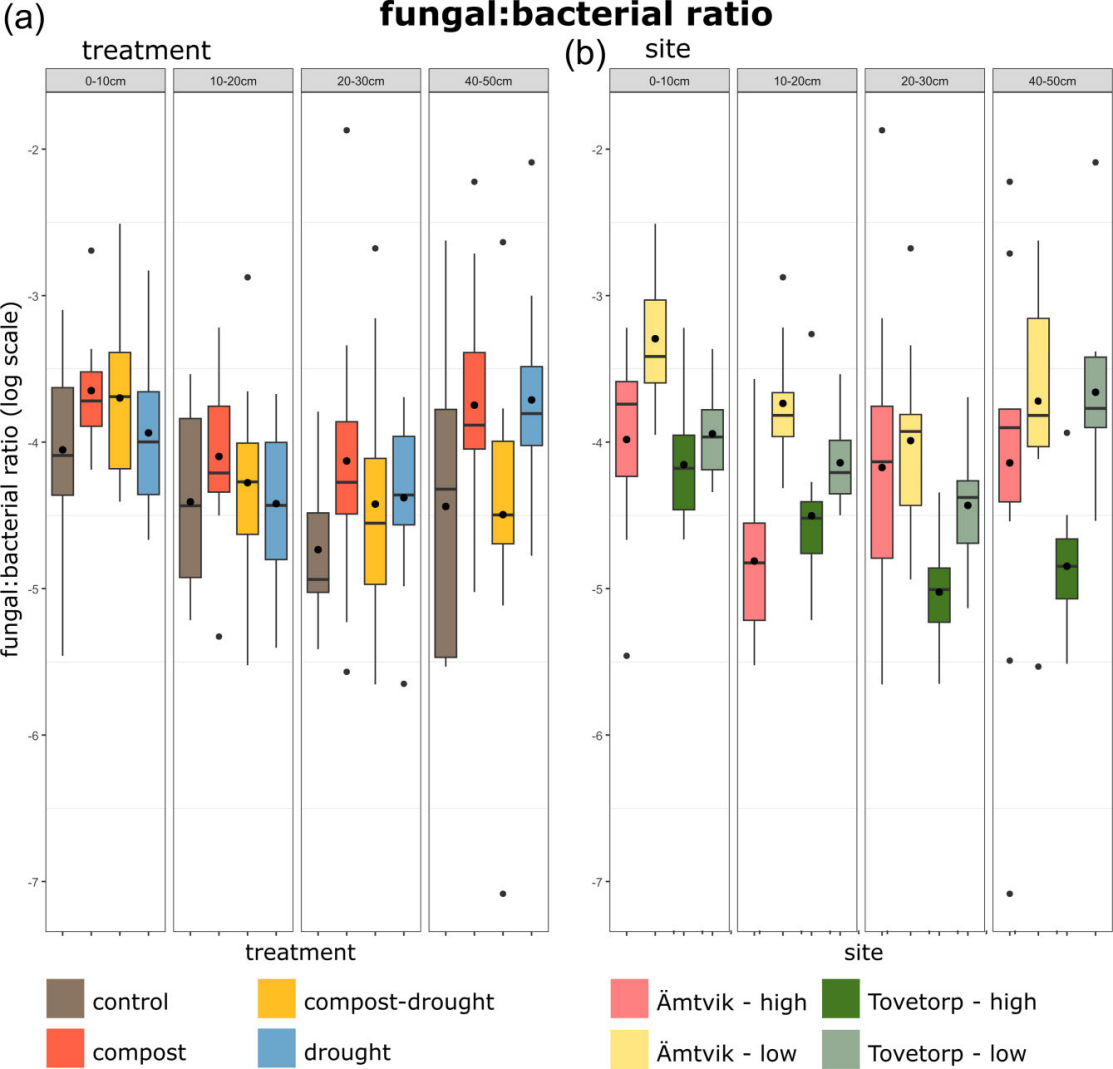
Fungal:bacterial ratio at different soil depths in response to drought and compost amendment (a) and at the four sampling locations (b) in south-east Sweden. Fungal and bacterial abundances were obtained through qPCR of fungal ITS and bacterial 16S DNA markers (copies mg^−1^ soil). Boxes show the mean (dot inside the box, *n* = 12), median (horizontal line), and interquantile range (IQR, colored box); whiskers extend to 1.5 IQR; and dots outside the boxes are outliers.

Fungal and bacterial abundances overall decreased with soil depth (F(3, 125.4) = 89.52, *P* < 0.01 and F(3, 126.67), *P* < 0.01, respectively; Supplementary Table S2). Fungal abundances increased significantly in the compost-amended plots compared to non-amended plots (F(1, 40.6) = 4.27, *P* = 0.04), with a significant compost × drought interaction (F(1, 40.7) = 5.28, *P* = 0.03), but neither drought nor compost affected bacterial abundance. Accordingly, the fungal:bacterial ratio was higher in compost-treated plots (F(1, 39) = 6.03, *P* = 0.01, Fig. 1) in the topsoil (0–10 cm), even though the variance of the fungal:bacterial ratio explained by random effects (site, R^2^ = 0.34) was higher than the variance attributed to treatments (R^2^ = 0.02).

**Figure 2.**
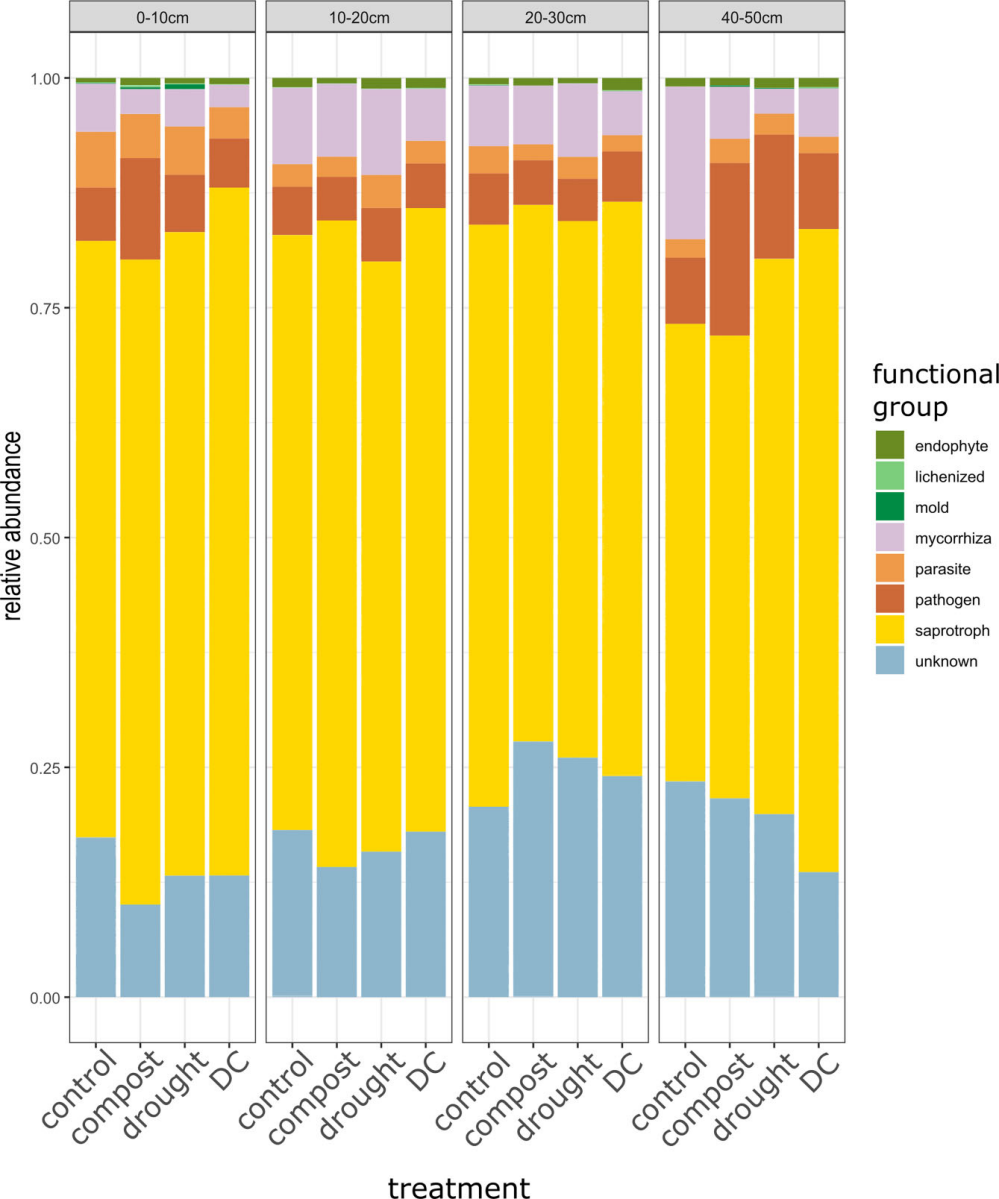
Relative abundances (*n* = 3) of fungal functional groups across treatments and depths. Stacked bars represent the relative abundances of the different functional groups as a fraction of total sequences. DC: combined drought and compost addition.

On average, the relative abundances of fungal functional groups (Fig. 2) were affected by compost (Wald = 4.761, *P* = 0.039). However, despite positive coefficients for saprotrophs and pathogens and negative coefficients for mycorrhizal fungi (not shown), this effect was not strong enough on any single group to yield statistically significant effects on specific functional groups (univariate tests, Supplementary Table S4). The relative abundance of fungal phyla in the topsoil was significantly affected by drought, both in drought-only plots and in combination with compost (multivariate tests, Wald = 4.926, *P* = 0.03 and Wald = 4.74, *P* = 0.038, respectively), but the effect was not detectable in any single group (Supplementary Table S4). Bacterial phyla were not affected by treatments at any depth.

**Figure 3.**
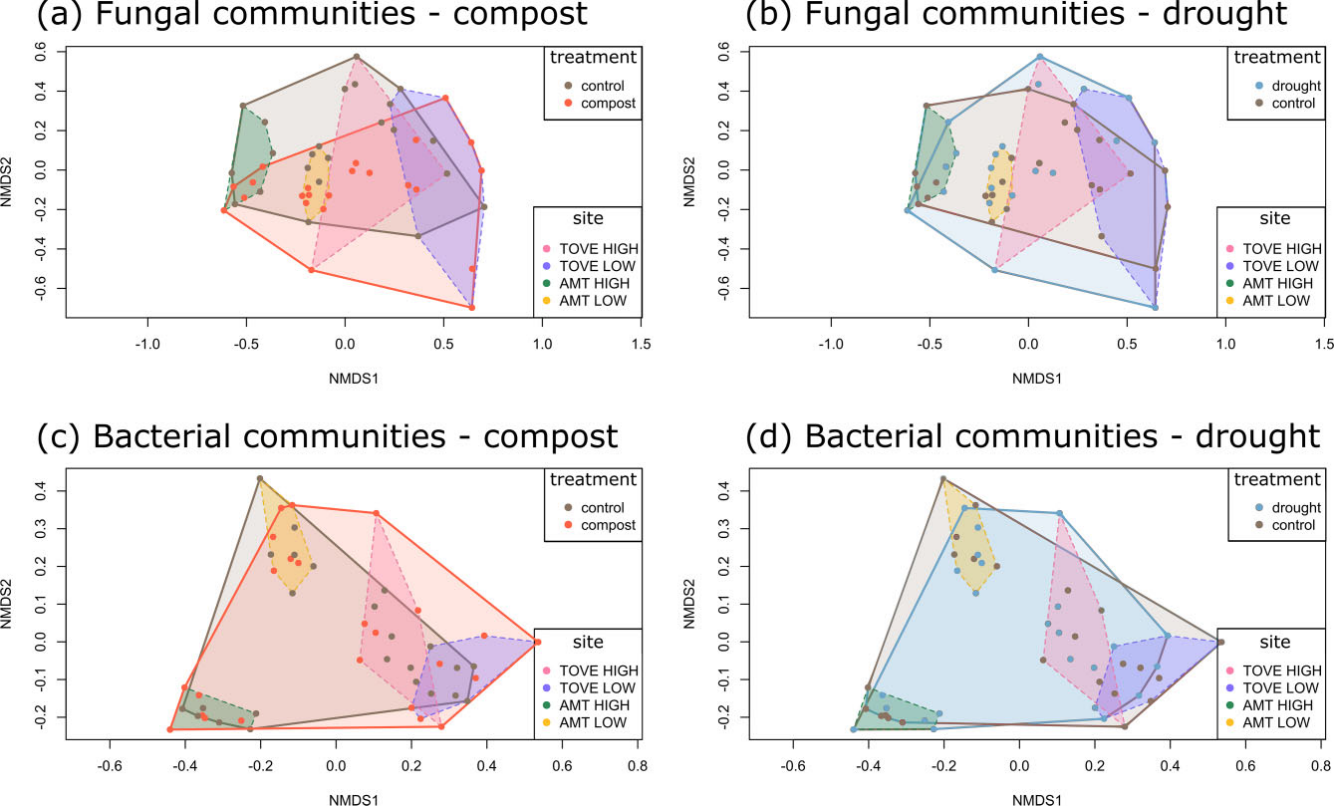
Effects of a compost treatment (a, c) and of a drought treatment (b, d) on the variation in soil fungal and bacterial community composition at the four grassland sites (Tovetorp high elevation, Tovetorp low elevation, Ämtvik high elevation, Ämtvik low elevation) in the top 10 cm of soil, visualized by NMDS ordinations, using a Bray–Curtis dissimilarity matrix. The data is based on PacBio sequencing of amplified ITS2 markers (fungi) and on Illumina MiSeq sequencing of amplified 16S rRNA gene markers (bacteria) in 48 soil plots.

For fungal communities, site explained the highest variance fraction, followed by depth, compost, and drought (Table 1). There was also a significant interaction between site and all other factors (depth, drought, and compost). When the topsoil was analyzed alone, site also explained the largest proportion of variation, followed by compost addition (Fig. 1a; Fig. 3a, b). For bacterial communities, site explained the largest proportion of variance, followed by depth, interaction between site and depth, interaction between site and drought, and drought. Only site had a significant effect on the bacterial community composition in the topsoil (Fig. 3c, d), explaining 48.2% of the total variance. Compost treatment led to significant dissimilarities between fungal communities, driven by changes in the topsoil (ANOSIM; all depths: R = 0.02, *P* = 0.03; 0–10 cm: R = 0.14, *P* < 0.01). This result was corroborated by a significant correlation between fungal community composition and soil organic C (Mantel; R = 0.09, *P* < 0.001), which was strongest in saprotrophs (R = 0.35, *P* < 0.001) and pathogens (R = 0.24, *P* < 0.001). However, the variability of fungal community composition was relatively high also within treatment groups.

**Table 1.** Results from PERMANOVA across all soil depths, for fungal and bacterial communities in two grasslands in south-east Sweden, each with an upper and a lower slope position.

Factor	Fungi			Bacteria		
	R^2^	F	*P*	R^2^	F	*P*
Drought	0.008	1.804	**0.006**	0.0052	1.933	**0.015**
Compost	0.014	3.222	**0.001**	0.003	1.337	0.164
Depth	0.058	4.499	**0.001**	0.139	17.429	**0.001**
Site	0.172	13.247	**0.001**	0.304	37.833	**0.001**
Drought*Site	0.023	1.809	**0.001**	0.011	1.387	**0.049**
Compost*Site	0.017	1.306	**0.017**	0.008	1.093	0.316
Depth*Site	0.06	1.553	**0.001**	0.101	4.207	**0.001**
Drought*Compost*Site	0.016	1.228	**0.048**	0.008	1.047	0.351
**Topsoil (0–10 cm)**						
Drought	0.0222	1.189	0.172	0.015	1.264	0.218
Compost	0.066	3.541	**0.001**	0.017	1.448	0.134
Site	0.221	3.950	**0.001**	0.475	13.246	**0.001**
Drought*Site	0.0556	0.994	0.496	0.038	1.073	0.340
Compost*Site	0.0503	0.9	0.764	0.028	0.792	0.789
Drought*Compost*Site	0.047	0.8477	0.887	0.031	0.86	0.683

### Indicator species analysis

In the top 10 cm, indicator species analysis identified 25 species as associated to the compost or drought treatments, or common to both. These 25 species constitute 5% of the total sequence counts across all samples, and 19% of the counts in samples within the first 10 cm of soil. Of these 24 species, 11 were identified as saprotrophs (62% of the total counts identified with the indicator species analysis, and 5% of all the saprotrophs detected in the study), and 6 were pathogens (29% of the total counts identified with the indicator species analysis, and 20% of all pathogens detected in the study). Taxonomic identifications are presented in Supplementary Table S3.

### Differences in microbial species richness and diversity between treatments

Fungal species richness and Shannon diversity were dependent on the interaction between compost addition and depth (F(6172) = 3.255; *P* < 0.05), where the compost addition lowered fungal species richness and diversity compared to control plots in the topsoil, and increased fungal richness and diversity in deeper soil. Soil organic C content correlated positively (*P* < 0.05) with the species richness of all fungi (r = 0.18), of saprotrophs (r = 0.31), and of parasitic fungi (r = 0.34), and with the diversity of parasitic fungi (r = 0.30). Soil moisture correlated negatively (*P* < 0.05) with the species richness of all fungi (r = −0.37), of saprotrophs (r = −0.36), and of pathogens (r = −0.30) and parasitic fungi (r = −0.43), and with the diversity of all fungi (r = −0.30), of saprotrophs (r = -0.17), and of pathogens (r = −0.37) and parasitic fungi (r = −0.39). Treatments had no significant effect on bacterial species richness or diversity, and there were no significant correlations of bacterial richness and diversity with soil organic C or soil moisture.

## Discussion

### Importance of spatial variation within microbial communities

Spatial heterogeneity, driven by factors like soil type, soil depth, vegetation, topography, and land management, plays a crucial role in microbial community composition, even at very small scales (Nunan et al. 2020). At our study sites, spatial factors such as grassland site and soil depth have already been shown to influence microbial communities (Guasconi et al. 2023). Consistent with previous results, we found that while the compost and drought treatments had some effects on microbial abundance, diversity, and community composition in the upper part of the soil profile, these effects were weaker than the spatial variation caused by grassland and slope position in all major microbial groups (Supplementary Table S4), as illustrated through the multivariate analysis (Fig. 3). These results are similar to the site-specific variation in drought effects on microbial communities also observed by Ochoa-Hueso et al. (2018). The compost-driven increase in soil organic C and drought-induced decline in soil moisture were consistent across sites, suggesting that the treatment effects *per se* were primarily additive, with consistent changes in soil conditions. However, despite these consistent effects on the soil environment, treatment effects on microbial communities were weak relative to other effects and varied by site, as indicated by significant treatment-by-site interactions. It is possible that this is due to the soil communities having been shaped by site-related environmental factors for far longer than the experimental treatments. In addition, the methods used in this study do not indicate which of the community members are active or dormant, or even dead. This could be of particular relevance to the qPCR analyses as well as for microbial community composition. These results stress the importance of considering a broader ecological context (e.g. land use and plant community composition) in field studies. This may allow differentiation of the relative contribution of experimental treatments (here a minor contribution) and landscape variability (large contribution) to variation in microbial community composition.

### Compost effects on microbial communities

The compost treatment increased fungal abundance and modified fungal community composition, including shifts in the relative abundance of functional groups, partly confirming our second hypothesis (that compost addition would increase microbial abundances with a relatively larger increase in saprotrophic and mycorrhizal fungi). Given that the compost had mostly decomposed by the time of sampling (Guasconi et al. 2025), the observed effects likely stem from the lasting influence of compost on soil organic C and nutrient availability—via plant communities or C pools with turnover times that are slower than that of the compost. These results align with findings by Lucas et al. (2014) and Bastida et al. (2017), who showed that organic amendments initially increased fungal abundance and altered community structure, which may have implications for the relative abundance of plant pathogen taxa or plant symbionts. Almost a third of the species counts identified as indicator species for the compost treatment belonged to known pathogens (Supplementary Table S3), and, while not statistically significant, there was a trend for increased relative abundance of this functional group in response to organic amendments. Because of the implications for plant productivity, this trend should be investigated with an appropriate study to assess whether the potential increase is due to a shift to more favorable conditions or direct inoculation through the compost addition. We also observed a positive correlation between fungal species richness and diversity and soil organic C with compost addition, in line with previous findings (Sun et al. 2016), and significant correlations between fungal community composition and soil organic C, which was strongest for saprotrophs. This could indicate that the compost amendment has provided a more diversified substrate for fungal growth, with effects still detectable after three growing seasons. However, we did not detect any significant effect on the species richness, diversity, or abundance of specific functional groups such as saprotrophs, which may indicate that over the long term the treatment does not directly affect ecosystem functions related to SOM decomposition. It is also worth noting that the dataset rarefaction, used to facilitate sample comparison, could potentially have affected the diversity and species richness analyses. Regarding the lack of detectable change in mycorrhizal abundance, it is important to note that ITS primers do not cover arbuscular mycorrhizal fungi to the same extent as they cover other fungal groups (Berruti et al. 2017), which could introduce bias in the results.

The compost treatment did not significantly affect bacterial abundance and diversity, and it affected bacterial community composition only in combination with drought. This is in contrast with previous studies showing that OA stimulated bacterial growth (Lucas et al. 2014, Bastida et al. 2017), diversity (Jaiswal et al. 2017, Bastida et al. 2017, Han et al. 2023), and altered bacterial community composition (Han et al. 2023) over short time scales (less than three months for all these studies). This inconsistency is likely due to a time lag between treatment and sampling, but could also be related to the quality of the OA and the amount of bioavailable C it contains. High content of bioavailable C can lead to stronger effects on microbial biomass and community structure (as in Lucas et al. 2014), whereas more recalcitrant C yields neutral effects. Overall, in our experiment, soil organic C management had a larger impact on fungal communities compared to bacterial communities.

### Drought effects on microbial communities

Overall, soil moisture reduction did not significantly affect microbial abundance or community composition. However, we observed a negative correlation between soil moisture and fungal richness and diversity. These results indicate a significant tolerance of microbial communities to drought, leading us to reject our initial hypothesis of drought-driven reductions in microbial abundance. One possible explanation is that the duration or intensity of our experiment may have limited the effects on the community composition. For instance, Canarini et al. (2021) found that drought impacts on microbial community composition became more pronounced with prolonged exposure, with significant effects observed only after 10 years of recurrent droughts. Therefore, our experiment may not have been long enough to capture these longer-term shifts in microbial community composition. Additionally, microbial biomass may increase under dry conditions due to reduced predation pressure, as soil fauna are more sensitive to drought than bacteria and fungi (Manzoni et al. 2012, Schaeffer et al. 2017). Overall, microbial communities in our experiment responded more to substrate availability than water availability. This suggests that increased organic inputs, achievable through land management when sources of organic amendments are available, may have a stronger impact on soil processes than rainfall variability and could therefore be a key to improve soil fertility and agricultural productivity, supporting SDG 2 Zero hunger.

Previous studies have also highlighted the drought tolerance of fungal communities (Canarini et al. 2017), due to a higher investment in the synthesis of storage compounds (Canarini et al. 2024). This may also be due to fungi’s ability to access a large soil volume through hyphal networks combined with more resistant cell walls (de Vries et al. 2012, Zhao et al. 2017). Conversely, bacteria may undergo larger shifts in community composition during drought, but they tend to recover quickly after rewetting, often returning to pre-drought conditions (Canarini et al. 2017, Li et al. 2023). Although drought represents a substantial stressor, microbial communities can enter dormancy or form spores to withstand dry conditions (Schimel et al. 2007). However, our qPCR-based estimates of abundance do not distinguish between active or dormant microorganisms, which could influence interpretations of microbial survival (Vos et al. 2013). The effects of drought on microbial enzymatic activity are also inconsistent: while some studies report reduced activity (Hueso et al. 2011, Franco-Andreu et al. 2017), others find no significant changes (Bastida et al. 2017). This variability may be attributed to microbial functional diversity, highlighting the importance of monitoring the impact of environmental changes on the relative abundance of taxa and functional groups. For example, drought may promote growth of saprotrophs (Lozano et al. 2021) while decreasing the relative abundance of mycorrhizal fungi (Ochoa-Hueso et al. 2018, Lozano et al. 2021). In our study, the presence of saprotrophs and pathogens among the indicator species for the treatments suggest that at species level there were some shifts in functional group composition under compost and drought. Changes in the balance of these groups may influence soil fertility, crop productivity, and carbon turnover, ultimately affecting agricultural sustainability and resilience to climate change. These differential responses may depend on changes in plant traits, in particular root traits (Lozano et al. 2021) such as RTD and SRL, which were observed in our experiment as well.

### Depth-dependence of treatment effects

Most treatment effects on microbial abundance, diversity, and community composition were significant only in the topsoil (0–10 cm), thereby confirming our third hypothesis of depth-dependence. The compost treatment significantly increased soil organic C content in the upper 10 cm of soil only, but the drought treatment significantly decreased soil moisture down to 30 cm depth. This suggests that microbial communities are less sensitive to soil moisture availability than originally expected. Soil depth is an additional spatial dimension for soil-dwelling organisms, and although microbial communities are most abundant in the topsoil, where most biological activity takes place because of the high organic matter input, the rhizosphere can extend far deeper. Microbial communities associated with deep roots in the subsoil can be large and diverse (Will et al. 2010, Li et al. 2014), contributing to nutrient cycling and carbon storage. This suggests that, on the time scale observed in this study, only a portion of the total soil microbial community—primarily those in the topsoil—was affected by changes in climate and environmental management. Therefore, considering soil depth is essential when interpreting treatment effects on microbial communities, as these may initially impact more accessible, surface-level communities rather than the deeper, subsoil communities, at least in permanent grasslands. However, since OA effects on soil properties can persist for several years (Sarker et al. 2022), it is not clear whether the effects from the management on soil microbial communities would be detectable also in deeper soil layers after longer time.

## Conclusions

The aim of this study was to test effects of growing season drought and compost amendments on soil microbial communities in varying grassland ecosystems. Contrary to expectations, we observed relatively limited microbial responses to these treatments. Growing season drought, despite a significant influence on soil moisture, exhibited only minor effects on microbial communities. Compost amendments, while increasing soil organic C content, affected mainly fungal abundance and community composition. This indicates that microbial communities are less sensitive to multi-year precipitation reduction and changes in land management (i.e. compost amendments) than what we had originally hypothesized. It also emphasizes the need to consider longer time perspectives, spatial variation at landscape scale, depth-dependent responses, and the specific pre-treatment traits of microbial communities. Overall, this understanding could inform sustainable land management practices that may increase the resistance of grassland ecosystems to environmental change.

## Supplementary Material

fnaf108_Supplemental_File

## Data Availability

The data concerning soil properties, soil moisture and root biomass and traits used in this study can be accessed via the Bolin Centre Database: https://doi.org/10.17043/guasconi-2025-soil-properties-1 (Guasconi et al. 2025).
