# Supplementary material for: Effects of compost amendments and experimental drought on grassland soil microbial communities
Source: FEMS Microbiol Lett. 2025 Oct 6;372:fnaf108. doi: 10.1093/femsle/fnaf108 (PMC12527337; doi:10.1093/femsle/fnaf108)
Supplement: fnaf108_Supplemental_File [file fnaf108_supplemental_file.docx]

# Supplementary material

The data relative to soil moisture, soil properties and root biomass and traits used in this paper can be accessed via the Bolin Centre Database: <https://bolin.su.se/data/guasconi-2025-soil-properties-1>

## Tables

Table S1. Fungal and bacterial phyla and fungal functional groups identified in the whole dataset

| Fungal Phylum | N of clusters | Percentage of total counts (%) |
| --- | --- | --- |
| Ascomycota | 580 | 47,49% |
| Basidiomycota | 314 | 43,03% |
| Calcarisporiellomycota | 1 | 0,02% |
| Chytridiomycota | 26 | 0,86% |
| Glomeromycota | 8 | 0,36% |
| Mortierellomycota | 57 | 4,40% |
| Mucoromycota | 28 | 0,56% |
| *unidentifed* | 139 | 3,27% |

| Fungal functional group | N of clusters | Percentage of total counts (%) |
| --- | --- | --- |
| AMF | 9 | 0.28% |
| ECM | 67 | 6.01% |
| Endophytes | 11 | 0.84% |
| Lichenized | 6 | 0.07% |
| Molds | 3 | 0.08% |
| Parasites | 65 | 3.03% |
| Pathogens | 79 | 7.29% |
| Saprotrophs | 469 | 63.92% |
| *Unidentified* | 433 | 18.48% |

| Bacterial Phylum | N of clusters | Percentage of total counts (%) |
| --- | --- | --- |
| Acidobacteriota | 828 | 14,846% |
| Actinobacteriota | 1089 | 29,912% |
| Armatimonadota | 55 | 0,088% |
| Bacteroidota | 236 | 0,749% |
| Bdellovibrionota | 54 | 0,033% |
| Chloroflexi | 908 | 14,744% |
| Crenarchaeota | 22 | 0,099% |
| Cyanobacteria | 18 | 0,016% |
| Dadabacteria | 1 | 0,001% |
| Deinococcota | 2 | 0,015% |
| Dependentiae | 42 | 0,039% |
| Desulfobacterota | 61 | 0,362% |
| Elusimicrobiota | 15 | 0,014% |
| Entotheonellaeota | 6 | 0,024% |
| Euryarchaeota | 1 | 0,0003% |
| Fibrobacterota | 3 | 0,004% |
| Firmicutes | 308 | 2,764% |
| GAL15 | 7 | 0,058% |
| Gemmatimonadota | 131 | 2,645% |
| Latescibacterota | 60 | 0,188% |
| MBNT15 | 12 | 0,054% |
| Methylomirabilota | 24 | 0,557% |
| Myxococcota | 363 | 1,548% |
| NB1.j | 3 | 0,004% |
| Nitrospirota | 20 | 0,198% |
| Patescibacteria | 161 | 0,168% |
| Planctomycetota | 420 | 0,967% |
| Proteobacteria | 863 | 14,034% |
| RCP2-54 | 22 | 0,245% |
| SAR324_clade(Marine_group_B) | 1 | 0,0001% |
| Sumerlaeota | 4 | 0,005% |
| Thermoplasmatota | 2 | 0,002% |
| Verrucomicrobiota | 409 | 15,040% |
| WPS-2 | 31 | 0,413% |
| WS2 | 1 | 0,001% |
| *unidentified* | 46 | 0,162% |

Table S2. F and P values for the aligned rank-transformed ANOVA on microbial abundance.

|  | Fungi | | Bacteria | | | |
| --- | --- | --- | --- | --- | --- | --- |
|  | F value | P value | F value | | P value | |
| drought | 1.00 | 0.32 | 0.31 | 0.58 | |  |
| compost | 4.27 | 0.04 | 0.68 | | 0.41 | |
| depth | 89.52 | < 0.01 | 105.89 | | < 0.01 | |
| drought:compost | 5.27 | 0.02 | 3.11 | | 0.08 | |
| drought:depth | 1.12 | 0.94 | 0.62 | | 0.59 | |
| compost:depth | 0.88 | 0.45 | 0.38 | | 0.76 | |
| drought:compost:depth | 2.42 | 0.06 | 2.26 | | 0.08 | |

| 0-10 cm depth | Fungi | | Bacteria | | | |
| --- | --- | --- | --- | --- | --- | --- |
|  | F value | P value | F value | | P value | |
| drought | 0.10 | 0.74 | 0.75 | 0.39 | |  |
| compost | 3.15 | 0.08 | 0.31 | | 0.57 | |
| drought:compost | 2.08 | 0.15 | 3.29 | | 0.07 | |

Table S3. Fungal taxa identified in the Indicator Species Analysis.

| treatment | Percentage of total counts (%) | Functional group | Lowest taxonomic level |
| --- | --- | --- | --- |
| Compost | 0.116 | pathogen | Didymella |
|  | 0.004 | *unknown* | *Unknown* |
|  | 0.005 | *unknown* | *Unknown* |
|  | 0.015 | saprotroph | Penicillium |
|  | 0.025 | pathogen | Typhula |
|  | 0.090 | pathogen | Fusarium |
|  | 0.016 | saprotroph | Pleosporomycetidae |
|  | 0.993 | pathogen | Cladosporium |
|  | 0.059 | saprotroph | Pucciniomycotina |
|  | 0.070 | pathogen | Alternaria |
| Drought | 0.234 | saprotroph | Hyaloscypha |
| Compost-Drought | 0.018 | parasite | Amesia |
|  | 0.013 | saprotroph | Goffeauzyma |
|  | 0.013 | saprotroph | Agaricales |
|  | 0.043 | *unknown* | Geastraceae |
| Compost + Compost-Drought | 2.714 | saprotroph | Monascaceae |
|  | 0.067 | saprotroph | Microascaceae |
|  | 0.059 | saprotroph | Aspergillus |
|  | 0.142 | *unknown* | *unknown* |
|  | 0.108 | saprotroph | Mycothermus |
|  | 0.023 | parasite | Amesia |
|  | 0.023 | saprotroph | Mrakiaceae |
|  | 0.068 | *unknown* | Chaetomiaceae |
|  | 0.211 | *unknown* | Rhizophydium |
|  | 0.151 | pathogen | Sarocladium |

Table S4. Results from *mvabund* analyses for fungal and bacterial phyla and for fungal functional groups

| *univariate* | | | |
| --- | --- | --- | --- |
| **Fungal Phylum** | **Factor** | **Dev** | **P-value** |
| Ascomycota | Treatment | 2.408 | 0.971 |
|  | Depth | 83.471 | **0.001** |
|  | Site | 44.18 | **0.003** |
|  | Treatment*depth | 13.043 | 0.710 |
|  | Treatment *site | 30.838 | **0.005** |
|  | Depth*site | 75.747 | **0.001** |
|  | Treatment *depth*site | 66.65 | 0.059 |
| Basidiomycota | Treatment | 2.559 | 0.971 |
|  | Depth | 42.226 | **0.001** |
|  | Site | 59.102 | **0.002** |
|  | Treatment*depth | 16.358 | 0.464 |
|  | Treatment *site | 20.748 | **0.020** |
|  | Depth*site | 85.435 | **0.001** |
|  | Treatment *depth*site | 61.46 | 0.077 |
| Calcarisporiellomycota | Treatment | 1.672 | 0.971 |
|  | Depth | 4.297 | 0.386 |
|  | Site | 16.738 | **0.007** |
|  | Treatment*depth | 11.823 | 0.710 |
|  | Treatment *site | 1.503 | 0.588 |
|  | Depth*site | 5.84 | 0.112 |
|  | Treatment *depth*site | 0.004 | 0.130 |
| Chytridiomycota | Treatment | 0.428 | 0.971 |
|  | Depth | 56.371 | **0.001** |
|  | Site | 2.088 | 0.657 |
|  | Treatment*depth | 43.76 | 0.032 |
|  | Treatment *site | 29.316 | **0.007** |
|  | Depth*site | 49.229 | **0.001** |
|  | Treatment *depth*site | 43.693 | 0.130 |
| Glomeromycota | Treatment | 0.952 | 0.971 |
|  | Depth | 16.303 | **0.001** |
|  | Site | 185.066 | **0.001** |
|  | Treatment*depth | 5.898 | 0.884 |
|  | Treatment *site | 6.663 | 0.588 |
|  | Depth*site | 26.9 | **0.004** |
|  | Treatment *depth*site | 36.69 | 0.130 |
| Mortierellomycota | Treatment | 8.728 | 0.489 |
|  | Depth | 68.46 | **0.001** |
|  | Site | 31.407 | **0.004** |
|  | Treatment*depth | 10.105 | 0.778 |
|  | Treatment *site | 27.45 | **0.007** |
|  | Depth*site | 62.027 | **0.001** |
|  | Treatment *depth*site | 57.972 | 0.086 |
| Mucoromycota | Treatment | 2.477 | 0.971 |
|  | Depth | 60.22 | **0.001** |
|  | Site | 35.935 | **0.003** |
|  | Treatment*depth | 21.578 | 0.216 |
|  | Treatment *site | 24.105 | **0.016** |
|  | Depth*site | 34.734 | **0.003** |
|  | Treatment *depth*site | 52.508 | 0.130 |
| *Unidentified* | Treatment | 1.514 | 0.971 |
|  | Depth | 71.34 | **0.001** |
|  | Site | 29.774 | **0.004** |
|  | Treatment*depth | 12.659 | 0.710 |
|  | Treatment *site | 41.912 | **0.003** |
|  | Depth*site | 61.93 | **0.001** |
|  | Treatment *depth*site | 59.892 | 0.077 |

| *univariate* | | | |
| --- | --- | --- | --- |
| **Fungal functional group** | **Factor** | **Dev** | **P-value** |
| endophyte | Treatment | 1.132 | 0.996 |
|  | Depth | 2.161 | 0.571 |
|  | Site | 56.679 | **0.001** |
|  | Treatment*depth | 10.644 | 0.942 |
|  | Treatment *site | 32.073 | **0.016** |
|  | Depth*site | 13.708 | 0.709 |
|  | Treatment *depth*site | 46.302 | 0.718 |
| lichenized | Treatment | 1.701 | 0.996 |
|  | Depth | 7.521 | 0.390 |
|  | Site | 16.657 | **0.004** |
|  | Treatment*depth | 9.186 | 0.966 |
|  | Treatment *site | 19.248 | 0.310 |
|  | Depth*site | 21.54 | 0.216 |
|  | Treatment *depth*site | 24.774 | 0.986 |
| mold | Treatment | 5.704 | 0.858 |
|  | Depth | 12.833 | 0.174 |
|  | Site | 9.262 | 0.052 |
|  | Treatment*depth | 11.521 | 0.922 |
|  | Treatment *site | 14.624 | 0.586 |
|  | Depth*site | 10.2 | 0.774 |
|  | Treatment *depth*site | 5.472 | 0.993 |
| mycorrhiza | Treatment | 5.95 | 0.858 |
|  | Depth | 6.378 | 0.398 |
|  | Site | 176.649 | **0.001** |
|  | Treatment*depth | 14.688 | 0.741 |
|  | Treatment *site | 17.782 | 0.360 |
|  | Depth*site | 11.641 | 0.774 |
|  | Treatment *depth*site | 19.244 | 0.993 |
| parasite | Treatment | 5.804 | 0.858 |
|  | Depth | 35.744 | **0.001** |
|  | Site | 21.961 | **0.001** |
|  | Treatment*depth | 7.019 | 0.970 |
|  | Treatment *site | 14.023 | 0.586 |
|  | Depth*site | 11.736 | 0.774 |
|  | Treatment *depth*site | 28.545 | 0.986 |
| pathogen | Treatment | 11.14 | 0.420 |
|  | Depth | 25.305 | **0.006** |
|  | Site | 52.635 | **0.001** |
|  | Treatment*depth | 17.805 | 0.546 |
|  | Treatment *site | 23.999 | 0.127 |
|  | Depth*site | 3.321 | 0.975 |
|  | Treatment *depth*site | 36.368 | 0.951 |
| saprotroph | Treatment | 0.888 | 0.996 |
|  | Depth | 10.726 | 0.257 |
|  | Site | 28.119 | **0.001** |
|  | Treatment*depth | 7.582 | 0.970 |
|  | Treatment *site | 9.229 | 0.778 |
|  | Depth*site | 22.83 | 0.167 |
|  | Treatment *depth*site | 21.784 | 0.993 |
| *Unidentified* | Treatment | 1.341 | 0.996 |
|  | Depth | 9.719 | 0.330 |
|  | Site | 52.941 | **0.001** |
|  | Treatment*depth | 5.691 | 0.970 |
|  | Treatment *site | 2.748 | 0.986 |
|  | Depth*site | 38.233 | **0.003** |
|  | Treatment *depth*site | 31.275 | 0.985 |

| *univariate* | | | |
| --- | --- | --- | --- |
| **Bacterial Phylum** | **Factor** | **Dev** | **P-value** |
| Acidobacteriota | Treatment | 0.756 | 0.999 |
|  | Depth | 64.34 | **0.001** |
|  | Site | 40.753 | **0.001** |
|  | Treatment*depth | 6.682 | 0.944 |
|  | Treatment *site | 13.285 | 0.883 |
|  | Depth*site | 90.089 | **0.004** |
|  | Treatment *depth*site | 46.812 | 0.653 |
| Actinobacteriota | Treatment | 1.398 | 0.998 |
|  | Depth | 68.532 | **0.001** |
|  | Site | 43.456 | **0.001** |
|  | Treatment*depth | 12.168 | 0.802 |
|  | Treatment *site | 11.627 | 0.941 |
|  | Depth*site | 100.595 | **0.004** |
|  | Treatment *depth*site | 36.08 | 0.771 |
| Armatimonadota | Treatment | 1.647 | 0.998 |
|  | Depth | 57.382 | **0.001** |
|  | Site | 58.097 | **0.001** |
|  | Treatment*depth | 10.574 | 0.802 |
|  | Treatment *site | 14.828 | 0.811 |
|  | Depth*site | 70.081 | **0.004** |
|  | Treatment *depth*site | 53.356 | 0.598 |
| Bacteroidota | Treatment | 1.756 | 0.998 |
|  | Depth | 63.511 | **0.001** |
|  | Site | 24.035 | **0.001** |
|  | Treatment*depth | 21.208 | 0.335 |
|  | Treatment *site | 9.682 | 0.951 |
|  | Depth*site | 97.468 | **0.004** |
|  | Treatment *depth*site | 52.431 | 0.606 |
| Bdellovibrionota | Treatment | 3.846 | 0.963 |
|  | Depth | 77.029 | **0.001** |
|  | Site | 16.517 | **0.015** |
|  | Treatment*depth | 12.402 | 0.795 |
|  | Treatment *site | 9.243 | 0.951 |
|  | Depth*site | 53.505 | **0.007** |
|  | Treatment *depth*site | 33.312 | 0.779 |
| Chloroflexi | Treatment | 1.197 | 0.999 |
|  | Depth | 30.507 | **0.001** |
|  | Site | 75.275 | **0.001** |
|  | Treatment*depth | 6.877 | 0.944 |
|  | Treatment *site | 7.092 | 0.951 |
|  | Depth*site | 121.394 | **0.004** |
|  | Treatment *depth*site | 34.185 | 0.771 |
| Crenarchaeota | Treatment | 0.919 | 0.999 |
|  | Depth | 25.599 | **0.001** |
|  | Site | 48.975 | **0.001** |
|  | Treatment*depth | 3.962 | 1.000 |
|  | Treatment *site | 7.851 | 0.951 |
|  | Depth*site | 93.723 | **0.004** |
|  | Treatment *depth*site | 70.866 | 0.511 |
| Cyanobacteria | Treatment | 1.811 | 0.998 |
|  | Depth | 20.528 | **0.001** |
|  | Site | 5.217 | 0.465 |
|  | Treatment*depth | 15.087 | 0.669 |
|  | Treatment *site | 15.516 | 0.786 |
|  | Depth*site | 34.868 | **0.014** |
|  | Treatment *depth*site | 49.909 | 0.653 |
| Dadabacteria | Treatment | 2.838 | 0.992 |
|  | Depth | 7.902 | 0.279 |
|  | Site | 14.801 | **0.020** |
|  | Treatment*depth | 12.033 | 0.802 |
|  | Treatment *site | 8.144 | 0.951 |
|  | Depth*site | 9.99 | 0.774 |
|  | Treatment *depth*site | 0.001 | 0.790 |
| Deinococcota | Treatment | 5.68 | 0.732 |
|  | Depth | 5.843 | 0.445 |
|  | Site | 3.832 | 0.653 |
|  | Treatment*depth | 0.005 | 1.000 |
|  | Treatment *site | 0.002 | 0.951 |
|  | Depth*site | 0 | 0.899 |
|  | Treatment *depth*site | 0 | 0.790 |
| Dependentiae | Treatment | 5.407 | 0.771 |
|  | Depth | 43.919 | **0.001** |
|  | Site | 28.847 | **0.001** |
|  | Treatment*depth | 11.304 | 0.802 |
|  | Treatment *site | 10.927 | 0.951 |
|  | Depth*site | 53.494 | **0.007** |
|  | Treatment *depth*site | 36.24 | 0.771 |
| Desulfobacterota | Treatment | 2.554 | 0.993 |
|  | Depth | 25.219 | **0.001** |
|  | Site | 46.826 | **0.001** |
|  | Treatment*depth | 6.644 | 0.944 |
|  | Treatment *site | 14.039 | 0.848 |
|  | Depth*site | 75.708 | **0.004** |
|  | Treatment *depth*site | 64.871 | 0.519 |
| Elusimicrobiota | Treatment | 0.897 | 0.999 |
|  | Depth | 45.871 | **0.001** |
|  | Site | 17.047 | **0.013** |
|  | Treatment*depth | 7.935 | 0.923 |
|  | Treatment *site | 13.392 | 0.883 |
|  | Depth*site | 43.391 | **0.008** |
|  | Treatment *depth*site | 70.904 | 0.511 |
| Entotheonellaeota | Treatment | 0.459 | 0.999 |
|  | Depth | 14.642 | **0.008** |
|  | Site | 15.971 | **0.015** |
|  | Treatment*depth | 42.288 | 0.089 |
|  | Treatment *site | 11.151 | 0.951 |
|  | Depth*site | 47.842 | **0.008** |
|  | Treatment *depth*site | 6.264 | 0.790 |
| Euryarchaeota | Treatment | 8.201 | 0.369 |
|  | Depth | 6.081 | 0.445 |
|  | Site | 9.801 | 0.111 |
|  | Treatment*depth | 0.356 | 1.000 |
|  | Treatment *site | 0.036 | 0.951 |
|  | Depth*site | 1.83 | 0.899 |
|  | Treatment *depth*site | 0.001 | 0.790 |
| Fibrobacterota | Treatment | 1.103 | 0.999 |
|  | Depth | 12.675 | **0.024** |
|  | Site | 3.806 | 0.653 |
|  | Treatment*depth | 17.827 | 0.448 |
|  | Treatment *site | 10.739 | 0.951 |
|  | Depth*site | 6.912 | 0.899 |
|  | Treatment *depth*site | 31.678 | 0.779 |
| Firmicutes | Treatment | 1.013 | 0.999 |
|  | Depth | 35.917 | **0.001** |
|  | Site | 44.5 | **0.001** |
|  | Treatment*depth | 2.609 | 1.000 |
|  | Treatment *site | 21.555 | 0.288 |
|  | Depth*site | 73.359 | **0.004** |
|  | Treatment *depth*site | 79.977 | 0.503 |
| GAL15 | Treatment | 1.696 | 0.998 |
|  | Depth | 7.745 | 0.279 |
|  | Site | 33.019 | **0.001** |
|  | Treatment*depth | 14.479 | 0.698 |
|  | Treatment *site | 9.177 | 0.951 |
|  | Depth*site | 25.923 | 0.101 |
|  | Treatment *depth*site | 68.25 | 0.519 |
| Gemmatimonadota | Treatment | 0.335 | 0.999 |
|  | Depth | 44.766 | **0.001** |
|  | Site | 62.995 | **0.001** |
|  | Treatment*depth | 12.392 | 0.795 |
|  | Treatment *site | 10.347 | 0.951 |
|  | Depth*site | 115.418 | **0.004** |
|  | Treatment *depth*site | 35.054 | 0.771 |
| Latescibacterota | Treatment | 2.124 | 0.998 |
|  | Depth | 35.106 | **0.001** |
|  | Site | 68.687 | **0.001** |
|  | Treatment*depth | 9.334 | 0.836 |
|  | Treatment *site | 9.987 | 0.951 |
|  | Depth*site | 78.067 | **0.004** |
|  | Treatment *depth*site | 38.977 | 0.712 |
| MBNT15 | Treatment | 1.769 | 0.998 |
|  | Depth | 28.288 | **0.001** |
|  | Site | 21.209 | **0.004** |
|  | Treatment*depth | 3.695 | 1.000 |
|  | Treatment *site | 3.3 | 0.951 |
|  | Depth*site | 73.534 | **0.004** |
|  | Treatment *depth*site | 22.039 | 0.790 |
| Methylomirabilota | Treatment | 0.542 | 0.999 |
|  | Depth | 14.819 | **0.007** |
|  | Site | 65.856 | **0.001** |
|  | Treatment*depth | 10.729 | 0.802 |
|  | Treatment *site | 8.67 | 0.951 |
|  | Depth*site | 101.825 | **0.004** |
|  | Treatment *depth*site | 42.695 | 0.653 |
| Myxococcota | Treatment | 1.089 | 0.999 |
|  | Depth | 56.305 | **0.001** |
|  | Site | 44.41 | **0.001** |
|  | Treatment*depth | 12.109 | 0.802 |
|  | Treatment *site | 10.701 | 0.951 |
|  | Depth*site | 97.874 | **0.004** |
|  | Treatment *depth*site | 69.475 | 0.514 |
| NB1.j | Treatment | 1.667 | 0.998 |
|  | Depth | 4.256 | 0.552 |
|  | Site | 32.031 | **0.001** |
|  | Treatment*depth | 34.914 | 0.097 |
|  | Treatment *site | 21.426 | 0.291 |
|  | Depth*site | 18.137 | 0.317 |
|  | Treatment *depth*site | 12.466 | 0.790 |
| Nitrospirota | Treatment | 3.081 | 0.987 |
|  | Depth | 12.169 | **0.033** |
|  | Site | 77.85 | **0.001** |
|  | Treatment*depth | 3.689 | 1.000 |
|  | Treatment *site | 8.415 | 0.951 |
|  | Depth*site | 69.093 | **0.004** |
|  | Treatment *depth*site | 46.125 | 0.653 |
| Patescibacteria | Treatment | 1.396 | 0.998 |
|  | Depth | 76.474 | **0.001** |
|  | Site | 18.673 | **0.009** |
|  | Treatment*depth | 13.483 | 0.770 |
|  | Treatment *site | 12.797 | 0.909 |
|  | Depth*site | 91.093 | **0.004** |
|  | Treatment *depth*site | 60.463 | 0.519 |
| Planctomycetota | Treatment | 0.696 | 0.999 |
|  | Depth | 58.478 | **0.001** |
|  | Site | 37.342 | **0.001** |
|  | Treatment*depth | 9.332 | 0.836 |
|  | Treatment *site | 10.407 | 0.951 |
|  | Depth*site | 105.319 | **0.004** |
|  | Treatment *depth*site | 81.256 | 0.502 |
| Proteobacteria | Treatment | 1.154 | 0.999 |
|  | Depth | 74.116 | **0.001** |
|  | Site | 31.418 | **0.001** |
|  | Treatment*depth | 9.13 | 0.836 |
|  | Treatment *site | 13.347 | 0.883 |
|  | Depth*site | 91.348 | **0.004** |
|  | Treatment *depth*site | 58.122 | 0.533 |
| RCP2.54 | Treatment | 1.077 | 0.999 |
|  | Depth | 42.657 | **0.001** |
|  | Site | 75.872 | **0.001** |
|  | Treatment*depth | 2.748 | 1.000 |
|  | Treatment *site | 16.243 | 0.711 |
|  | Depth*site | 86.267 | **0.004** |
|  | Treatment *depth*site | 62.258 | 0.519 |
| SAR324_clade.Marine_group_B. | Treatment | 2.871 | 0.992 |
|  | Depth | 5.715 | 0.445 |
|  | Site | 6.543 | 0.366 |
|  | Treatment*depth | 0.005 | 1.000 |
|  | Treatment *site | 0.003 | 0.951 |
|  | Depth*site | 0.002 | 0.899 |
|  | Treatment *depth*site | 0 | 0.790 |
| Sumerlaeota | Treatment | 0.565 | 0.999 |
|  | Depth | 22.707 | **0.001** |
|  | Site | 0.73 | 0.813 |
|  | Treatment*depth | 13.262 | 0.770 |
|  | Treatment *site | 12.014 | 0.940 |
|  | Depth*site | 4.841 | 0.899 |
|  | Treatment *depth*site | 44.178 | 0.653 |
| Thermoplasmatota | Treatment | 2.037 | 0.998 |
|  | Depth | 4.498 | 0.552 |
|  | Site | 16.888 | **0.015** |
|  | Treatment*depth | 12.156 | 0.802 |
|  | Treatment *site | 2.205 | 0.951 |
|  | Depth*site | 0.001 | 0.899 |
|  | Treatment *depth*site | 0.002 | 0.790 |
| Verrucomicrobiota | Treatment | 1.308 | 0.998 |
|  | Depth | 54.177 | **0.001** |
|  | Site | 41.654 | **0.001** |
|  | Treatment*depth | 5.369 | 0.984 |
|  | Treatment *site | 10.816 | 0.951 |
|  | Depth*site | 89.543 | **0.004** |
|  | Treatment *depth*site | 42.342 | 0.653 |
| WPS.2 | Treatment | 1.187 | 0.999 |
|  | Depth | 51.147 | **0.001** |
|  | Site | 144.77 | **0.001** |
|  | Treatment*depth | 1.807 | 1.000 |
|  | Treatment *site | 14.423 | 0.830 |
|  | Depth*site | 47.308 | **0.008** |
|  | Treatment *depth*site | 44.874 | 0.653 |
| WS2 | Treatment | 3.319 | 0.980 |
|  | Depth | 4.403 | 0.552 |
|  | Site | 2.434 | 0.666 |
|  | Treatment*depth | 20.612 | 0.367 |
|  | Treatment *site | 8.475 | 0.951 |
|  | Depth*site | 20.34 | 0.317 |
|  | Treatment *depth*site | 10.127 | 0.790 |
| *unidentified* | Treatment | 0.714 | 0.999 |
|  | Depth | 40.898 | **0.001** |
|  | Site | 18.267 | **0.011** |
|  | Treatment*depth | 7.323 | 0.936 |
|  | Treatment *site | 10.917 | 0.951 |
|  | Depth*site | 115.038 | **0.004** |
|  | Treatment *depth*site | 61.343 | 0.519 |

## Figures

a) fungi


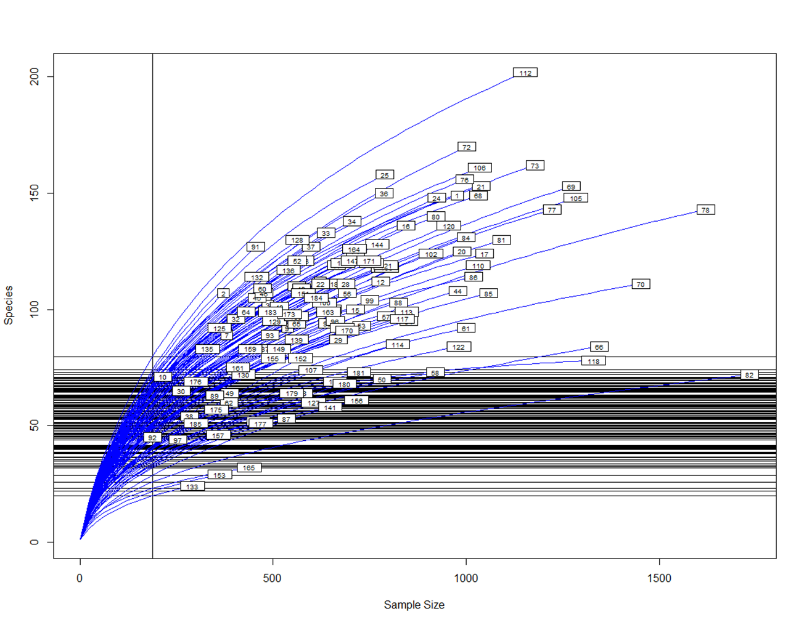


b) bacteria


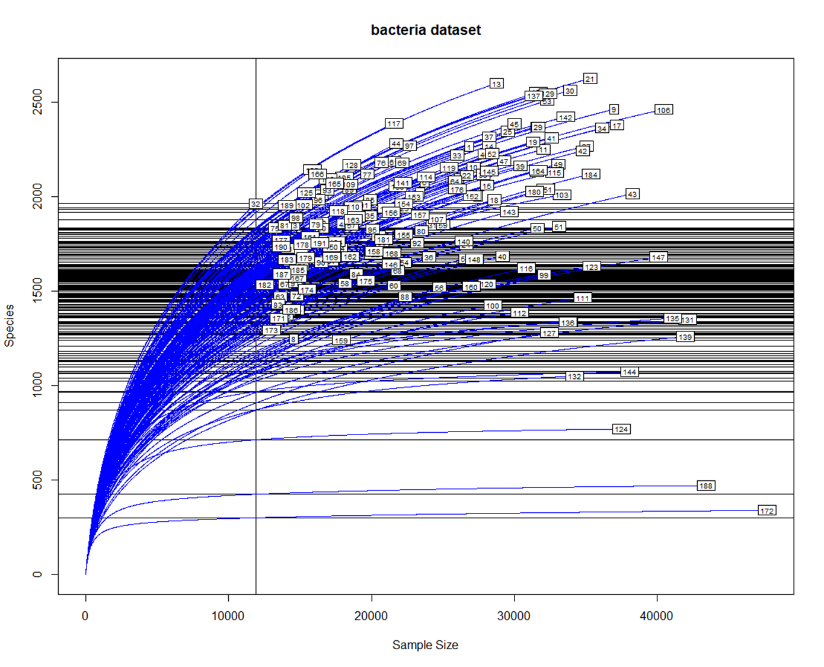


Figure S1. Species accumulation curves for (a) fungal OTUs and (b) bacterial OTUs for each sample.


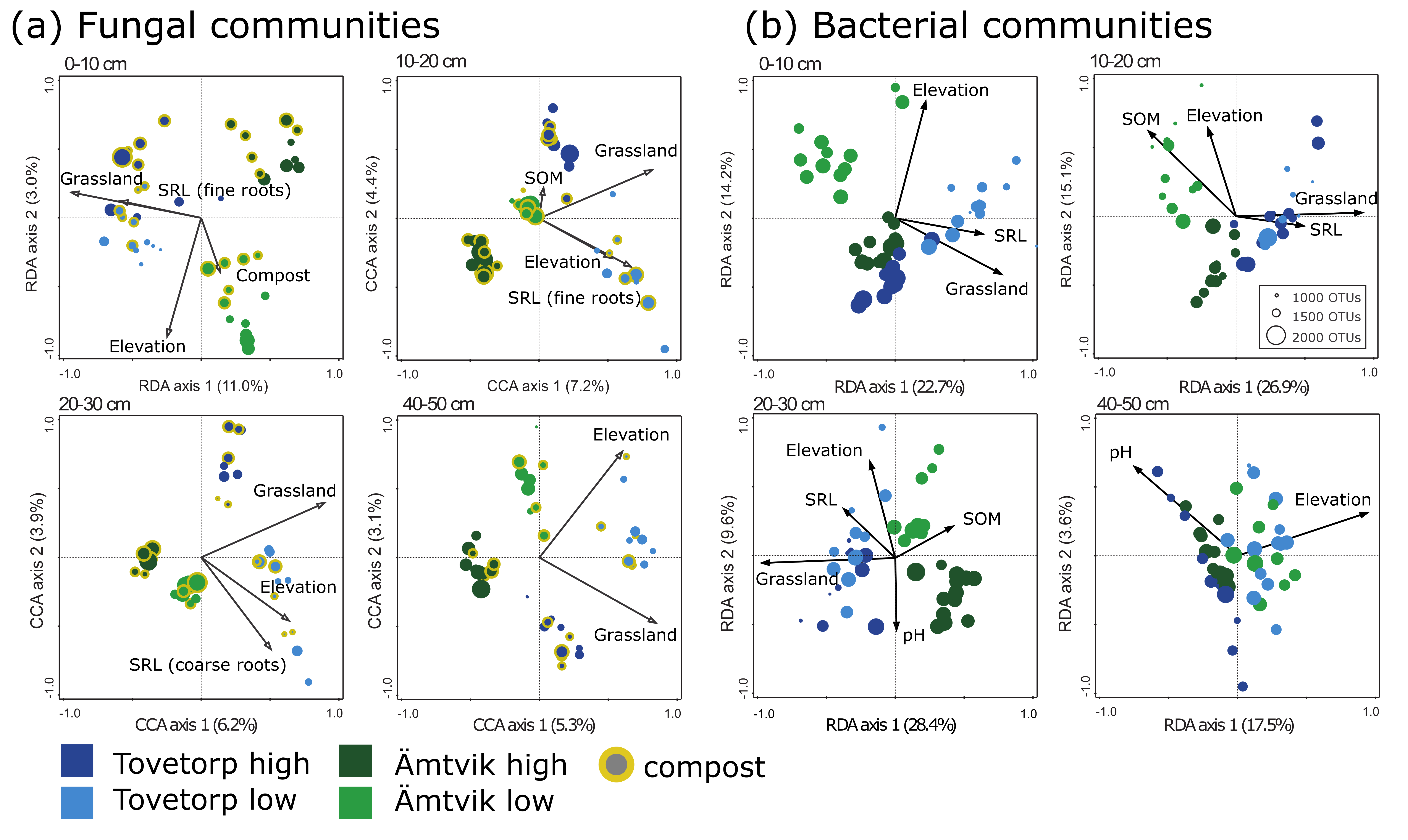


Figure S2. Effects of a compost treatment (golden ring) on the variation in soil fungal and bacterial community composition in four grassland sites (Tovetorp (blue) and Ämtvik (green), low (light shades) and high (darker shades) elevation) at four different soil depths, visualized by sample plots of redundancy analyses (RDA; fungi 0-10 cm and bacteria) or canonical correspondence analyses (CCA; fungi 10-20 cm, 20-30 cm and 40-50 cm) based on PacBio sequencing of amplified ITS2 markers (fungi) and on Illumina MiSeq sequencing of amplified 16S rRNA gene markers (bacteria) in 48 soil plots. Dot size represents the number of OTU in each sample. Soil depths are 0-10 cm (top left), 10-20 cm (top right), 20-30 cm (lower left) and 40-50 cm (lower right). The RDAs included a total of 1138 identified fungal OTUs, data not rarefied, and 6183 identified bacterial OTUs, data rarefied. For fungi, black vectors represent constraining variables and indicate direction and degree of correlation between RDA axes and grassland, slope position, compost treatments, specific root length (fine roots), and SOM. Axes 1 and 2 together explained 15.3, 9.3, 9.5 and 8.4% of the total variation for 0-10 cm, 10-20 cm, 20-30 cm and 40-50 cm, respectively, when accounting for sequencing depth in each sample. Total variation was 41131, 6.6, 8.5 and 8.9 for respective soil depth. For bacteria, vectors represent constraining variables and indicate direction and degree of correlation between RDA axes and grassland, slope position, specific root length (SRL) of fine roots, SOM and pH. Axes 1 and 2 together explained 36.8, 42.0, 37.9 and 21.1% of the total variation for 0-10 cm, 10-20 cm, 20-30 cm and 40-50 cm, respectively, when accounting for sequencing depth in each sample. The total variation was 0.068, 0.087, 0.17 and 0.39 for respective soil depth.


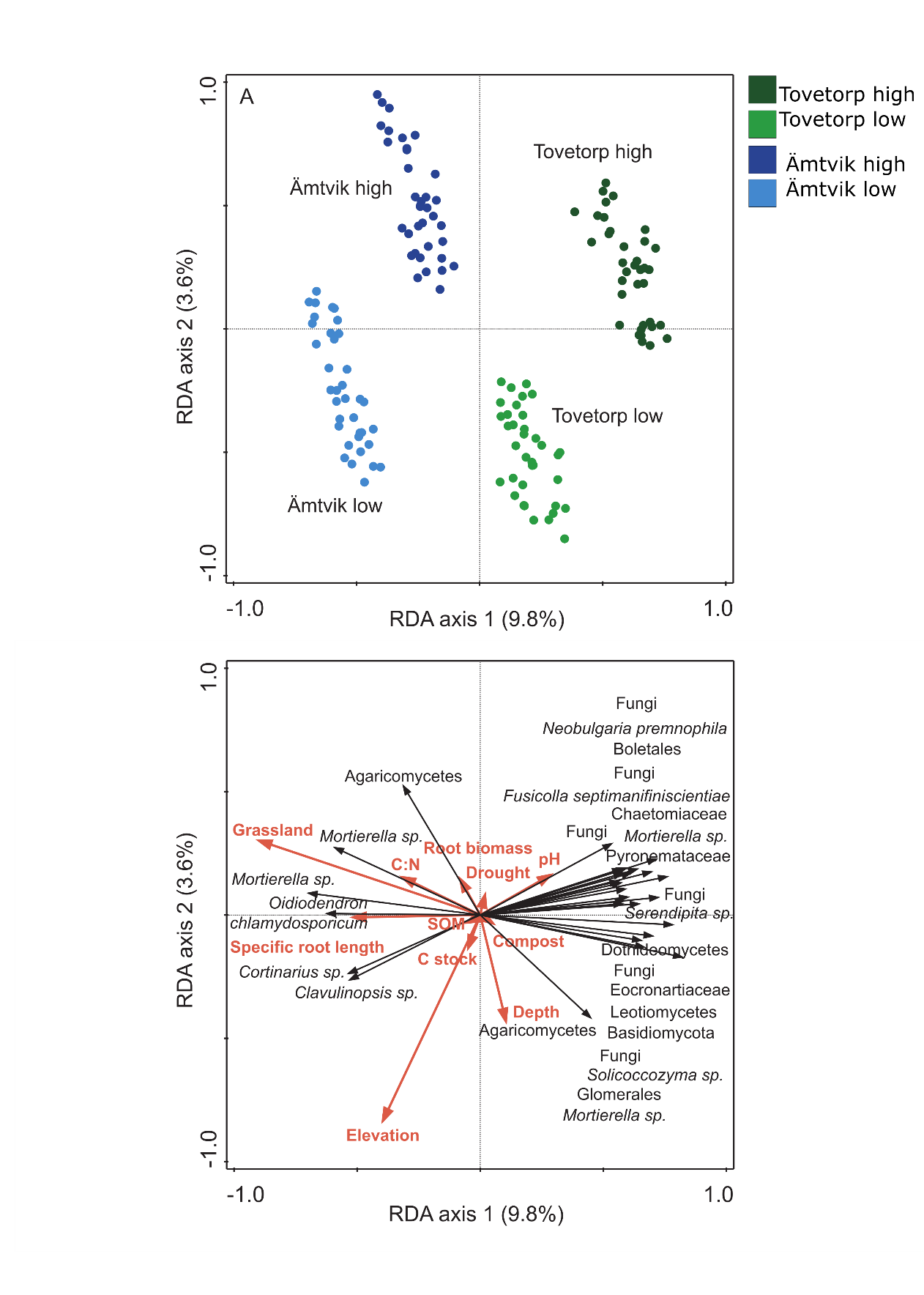


Figure S3. Redundancy analyses (RDA) of compost and drought treatment effects on fungal communities in soil profiles (0-30 cm) at two grassland sites with two catenary positions in south-central Sweden (n=144). Fungal community composition is visualized by species plots of RDAs based on PacBio sequencing of amplified ITS2 markers. The RDAs included 1063 identified fungal OTUs (data not rarefied). Red vectors represent constraining variables, and indicate direction and degree of correlation between RDA axes and categorical variables grassland, catenary position, depth, compost and drought, and the plant and soil variables root biomass, specific root length, C:N ratio, pH, soil organic matter (SOM) and C stock. Only the 30 most abundant OTU are shown. Axes 1 and 2 for fungi explained 13.4 % together of the total variation of 130466.5 when accounting for sequencing depth in each sample. The adjusted explained variation is 17.6% for categorical, plant and soil variables. Seven missing samples and 6 missing variables were replaced by the mean number of counts or measured variable based on the two replicates within the same treatment. The effect of treatments and variables was tested by restricted Monte Carlo permutations accounting for dependency of communities in soil depths from the same plot, and by using forward selection of explanatory variables (False discovery rate correction). Sequencing depth was included as a covariate.
